# Two New Iridoid Glycosides from the Root Barks of *Sambucus williamsii* Hance

**DOI:** 10.3390/molecules16053869

**Published:** 2011-05-09

**Authors:** Zhen-Yue Wang, Hua Han, Bing-You Yang, Yong-Gang Xia, Hai-Xue Kuang

**Affiliations:** Key Laboratory of Chinese Materia Medica, Heilongjiang University of Chinese Medicine, Ministry of Education, Harbin 150040, China

**Keywords:** *Sambucus williamsii* Hance, root barks, iridoid glycosides

## Abstract

Chemical investigation of the ethanol extract of the root barks of *Sambucus williamsii* Hance collected in the Heilongjiang province of China resulted in the isolation of two new iridoid glycosides, williamsoside A (**1**) and williamsoside B (**2**). Their structures were elucidated on the basis of extensive spectroscopic analysis (1D, 2D-NMR and HRESIMS) and chemical studies. Iridoid glycosides have for a long time been considered as characteristic ingredients of *S. williamsii*. However, the presence of iridoid glycosides with apiofuranosyl moieties in *S. williamsii* is reported for the first time in this study.

## 1. Introduction

*Sambucus williamsii* Hance, a tree widely distributed in China, belongs to the family Adoxaceae [1]. *S. williamsii* is also called in Chinese “Jie-gu-mu”, “Da-jie-gu-dan”, “Gong-lao-dao”, *etc.* [2]. The root bark of this plant is a folk medicine with a long history of use in China for the treatment of fractures and osteoporosis [2]. Phytochemical studies showed that triterpenoids, flavonoids, lignans and the iridoid morroniside are major constituents of *S. williamsii* [3]. In our present work, investigation of its ethanol extract led to the isolation of two new iridoid glycosides. In this paper, we present the isolation and structural characterization of the two new iridoid glycosides on the basis of the interpretation of spectral data, including 1D, 2D NMR and HRESIMS data.

## 2. Results and Discussion

Compound **1** was obtained as a white amorphous powder and showed positive results for the Molisch reagent, which was considered to be indicative of an iridoid glycoside. Its molecular formula was established as C_22_H_34_O_13_ by the positive HRESIMS data, indicating six degrees of unsaturation.

The ^1^H-NMR spectrum of **1** (Table 1) showed the two characteristic signals of the common iridoid compounds, namely a proton at *δ* 5.11 (1H, d, *J* = 6.2 Hz) and a singlet at *δ* 7.41 (1H) corresponding to the C-1 and C-3 protons, respectively. In addition, the signals at *δ* 3.68 (3H, s) and 1.09 (3H, d, *J* = 6.7 Hz) were attributed to Me-12 and Me-10, respectively. The typical downfield signals at *δ* 4.64 (1H, d, *J* = 7.9 Hz), 4.99 (1H, d, *J* = 2.3 Hz) were assigned to the anomeric H-atom of the *β*-glucopyranosyl and *α*-apiofuranosyl moieties according to their coupling constants and splitting patterns [4].

**Table 1:** ^1^H and ^13^C-NMR data of **1 **and **2** in CD_3_OD at 400 MHz and 100 MHz, *J* in Hz.

No.	1		2	
	*δ*_H_	*δ*_C_	*δ*_H_	*δ*_C_
1	5.11 (1H, d, *J* = 6.2)	98.2	5.15 (1H, d, *J* = 5.5)	98.2
3	7.41 (1H, s)	152.8	7.39 (1H, d, *J* = 1.2)	152.3
4		112.8		113.8
5	2.87 (1H, dd, *J* = 7.8, 15.7)	35.5	3.11 (1H, dd, *J* = 7.9, 16.2)	32.4
6	2.20 (1H, m), 1.35 (1H, m)	33.6	1.60 (1H, m), 2.23(1H, m)	42.8
7	1.90 (1H, m), 1.89 (1H, m)	34.1	4.05 (1H,dd, *J*= 4.4, 4.0)	75.0
8	1.99 (1H, q, *J* = 6.9, 7.3)	36.6	1.89 (1H, m)	42.4
9	1.73 (1H, m)	48.4	1.99 (1H, dt, *J* = 3.8, 5.0)	46.5
10	1.09 (3H, d, *J* = 6.7)	21.0	1.10 (3H, d, *J* = 6.8)	13.7
11		169.7		169.5
12	3.68 (3H, s)	51.7	3.68(3H, s)	51.6
1’	4.64 (1H, d, *J* = 7.9)	100.4	4.62 (1H, d, *J* = 7.9)	100.3
2’	3.19 (1H, m)	74.7	3.19 (1H, m)	74.7
3’	3.35 (1H, m)	77.9	3.34 (1H, m)	77.9
4’	3.29 (1H, m)	71.5	3.26 (1H, m)	71.6
5’	3.42 (1H, m)	77.2	3.42 (1H, m)	77.2
6’	3.68 (1H, m), 3.97 (1H, m)	68.4	3.62 (1H,m), 3.98 (1H,m)	68.5
1”	4.99 (1H, d, *J* = 2.3)	110.9	5.00 (1H, d, *J* = 2.4)	110.9
2”	3.88 (1H, m)	77.9	3.88(1H, m)	77.9
3”		80.5		80.5
4”	3.95 (1H, dd, *J* = 9.6)	75.0	3.75(1H, dd, *J* = 9.6)	75.0
	3.75(1H, dd, *J* = 9.6)		3.94(1H, dd, *J* = 9.6)	
5”	3.55(1H, s)	65.6	3.55 (2H, s)	65.5

**Figure 1:**
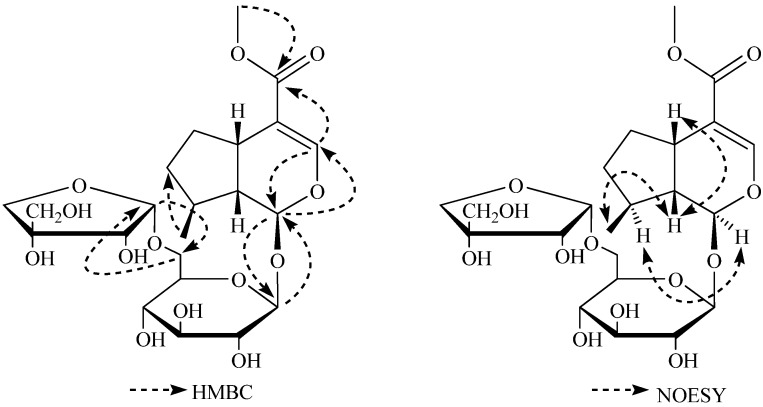
Key HMBC and NOESY correlations of **1**.

The ^13^C-NMR spectrum of **1** (Table 1) showed resonances for 22 C-atoms, including two quaternary carbons (*δ* 112.8, 169.7), five methines (*δ* 98.2, 152.8, 35.5, 36.6, 48.4), two methylenes (*δ* 33.6, 34.1), and two methyls (*δ* 21.0, 51.7) belonging to the aglycone moiety, a glucopyranosyl group (*δ* 100.4, 74.7, 77.9, 71.5, 77.2, 68.4), and an apiofuranosyl group (*δ* 110.9, 77.9, 80.5, 75.0, 65.6). C-1 that was connected with C-3 through an oxygen atom on the basis of the chemical shifts of C-1(*δ* 98.2) and C-3 (*δ* 152.8), which was also confirmed by the HMBC correlations from H-3/C-1 and H-1/C-3. The HMBC spectrum was used to elucidate the connection of different structural fragments, as well as to confirm the above assignments. In this spectrum, other key long-range correlations were observed between Me-10/C-7, C-8 and C-9, and between Me-12/C-11 (Figure 1). The connection positions of the glucopyranosyl and apiofuranosyl groups in **1** were established unambiguously by a HMBC experiment in which long-range correlations between H-1’/C-1, H-1/C-1’, H-1”/C-6’, and H-6’/C-1”.

**Figure 2:**
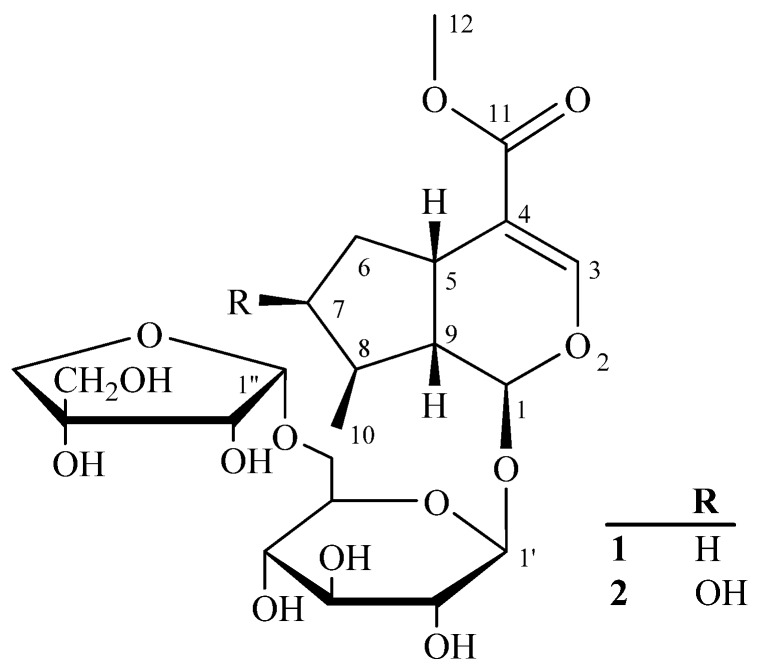
Structures of **1** and **2**.

Since the stereochemistry of the three asymmetric centers (C-1, C-5, and C-9) was the same in practically all iridoids identified hitherto [5,6], the C-1 oxygen atom, H-5 and H-9 were assigned the *β-*orientation in the iridoid skeleton. The stereo configuration of Me-10 was determined as a *β-*orientation on the basis of the key NOESY correlations between Me-10/H-9, H-9/H-5, and H-8/H-1 (Figure 2). Thus, the structure of **1** was identified to be 6’-apiosyldeoxyloganin, with the structure shown in Figure 2, and it was named williamsoside A.

Compound **2** was obtained as a white amorphous powder and showed positive results for the Molisch reagent, which was also considered indicative of another iridoid glycoside. Its molecular formula was established as C_22_H_34_O_14_ by the positive HRESIMS, indicating six degrees of unsaturation. The ^1^H-NMR spectrum of **2** showed distinct resemblance to that of **1**. The only notable difference was the change in the H-7 signal, which appeared as a double doublet at *δ* 4.05 (1H, dd, *J* = 4.4, 4.0 Hz), indicating that C-7 was substituted by a hydroxyl group. The ^13^C-NMR (DEPT) spectrum showed an additional downfield C-atom signal at *δ* C 75.0 in **2**, which was affirmatively assigned to the C-7 carbon. The stereo configuration of the hydroxyl group at C-7 and methyl group at C-8 was determined to be the *β-*orientation on the basis of the obvious NOESY correlations between H-7/H-8, H-8/H-1, Me-10/H-9, and H-9/H-5. On the basis of above data, the structure of **2** was identified to be as shown in Figure 2, and it was named williamsoside B.

## 3. Experimental

### 3.1. General

IR spectra were recorded on a Shimadzu FTIR-8400S spectrometer. NMR spectra were recorded on a Bruker DPX 400 NMR instrument (at 400 MHz for ^1^H-NMR and 100 MHz for ^13^C-NMR). Chemical shifts are given as *δ* values with reference to tetramethylsilane (TMS) used as internal standard, and coupling constants are given in Hz. HRESIMS were carried out on Waters Xevo QTOF mass spectrometer. Preparative HPLC (Waters, Delta 600-2487) was performed on a Hypersil-ODS II (10 m, 20 × 300 mm, Yilite, Dalian, China).

### 3.2. Plant Material

The root barks of *S. williamsii* were collected in August 2008 from the Fangzheng district, Heilongjiang Province, China, and identified by the author Zhen-Yue Wang. A voucher specimen (20080079) has been deposited at Heilongjiang University of Chinese Medicine, Harbin, China.

### 3.3. Extraction and Isolation

The dried root barks (5.0 kg) of *S. williamsii* were extracted with 95% EtOH under reflux (2 × 10 L) for 2 h (each time), and the combined soln. was filtered and concentrated under vacuum to an oily residue, which was suspended in H_2_O. The suspension was passed through AB-8 crosslinked polystyrene, and sequentially eluted with H_2_O, 50% EtOH, and 95% EtOH, respectively. The 50% EtOH elution fraction was concentrated under vacuum to yield a residue (52.0 g), which was subjected to silica gel column and eluted successively with CHCl_3_/MeOH (15:1→1:1) to give 10 fractions (Fraction 1–10). Fraction 7 (5 g) was further separated by ODS column to afford 9 sub-fractions A_1_–A_9_. The sub-fraction A_9_ was subjected to preparative HPLC (Hypersil-ODS Π column) eluted with MeOH/H_2_O (2:3) to afford compounds **1** (28 mg) and **2** (49 mg).

*Williamsoside A* (**1**): White amorphous powder, [α]^25^_D_ = −27.0 (c = 0.1, MeOH). IR (KBr): *ν* = 3303, 2945, 2831, 1448, 1417, 1114, 1035, 659 cm^−1^. HRESIMS (positive): *m/z* = 507.2057 (calc. for C_22_H_3__5_O_13_, 507.2028, [M + H]^+^), 524.2352 (calc. for C_22_H_38_NO_13_, 524.2343, [M + NH_4_]^+^), 529.1880 (calc. for C_22_H_3__4_NaO_13_, 529.1897, [M + Na]^+^) and 545.1628 (calc. for C_22_H_3__4_KO_13_, 545.1636, [M + K]^+^). ^1^H and ^13^C-NMR: see Table 1.

*Williamsoside B* (**2**): White amorphous powder, [α]^25^_D_ = −16.0 (c = 0.1, MeOH). IR (KBr): *ν* = 3312, 2945, 2833, 1447, 1419, 1113, 1035, 660 cm^−1^. HRESIMS (positive): *m/z* = 523.2002 (calc. for C_22_H_35_O_14_, 523.2027, [M + H]^+^), 540.2271 (calc. for C_22_H_38_NO_14_, 540.2292, [M + NH_4_]^+^), and 545.1849 (calc. for C_22_H_34_NaO_14_, 545.1846, [M + Na]^+^). ^1^H and ^13^C-NMR: see Table 1.

*Acid Hydrolysis of* **1** *and* **2**. To a solution of **1** and **2** (each, 250 μg) in MeOH (1 mL) was added 5% H_2_SO_4_ (1 mL) and the mixture was refluxed for 8 h. The reaction mixture was then neutralized with saturated sodium carbonate and extracted with ethyl acetate (EtOAc, 2 × 5 mL) to give an aqueous fraction containing sugars and an EtOAc fraction containing the aglycone part. The aqueous phase was concentrated and compared with standard sugars using the TLC eluent systems EtOAc/*n*-butanol/water (2:7:1) and CH_2_Cl_2_/MeOH/water (10:6:1) [7,8,9]; the two sugars were thus identified as apiose and glucose.

## 4. Conclusions

Iridoid glycosides represent a large group of cyclopentano[*c*]pyran monoterpenoids which have been reported to be associated with diverse biological activities including choleretic, purgative, liver protective, vasoconstrictive, antimicrobial, analgesic, antitumor, sedative and anti-inflammatory properties [10]. As a part of our chemical investigation on *S. williamsii*, we have isolated two new iridoid glycosides containing a 6’-apiofuranosyl moiety. Their structures were established on the basis of spectroscopic evidence. This is the first time iridoid glycosides with apiofuranosyl moieties have been reported in this species.
